# Proanthocyanidin from Grape Seed Extracts Protects Indomethacin-Induced Small Intestinal Mucosal Injury

**DOI:** 10.1155/2014/618068

**Published:** 2014-04-27

**Authors:** Dae Young Cheung, Jin Il Kim, Soo-Heon Park, Jae Kwang Kim

**Affiliations:** The Department of Internal Medicine, The Catholic University of Korea College of Medicine, St. Mary's Hospital, Yeongdeungpo-gu 63-ro 10, Seoul 150-713, Republic of Korea

## Abstract

Proanthocyanidin (grape seed proanthocyanidin extracts, GSPEs) is an antioxidant and scavenges free radicals. Excessive oxidative stress and free radical production are major components in the pathogenesis of NSAID-induced small intestinal injury. We investigated the effect of GSPEs on indomethacin-induced intestinal mucosal injury in the rat. Rats were allocated into four groups: the null control group, the indomethacin control group, the low-dose GSPEs group, and the high-dose GSPEs group. GSPEs were administered for 4 days. Then indomethacin and GSPEs were coadministered for the following 2 days by oral route. The dose of indomethacin was 200 mg/Kg. The doses of GSPEs were 100 mg/Kg for low-dose group and 300 mg/Kg for high-dose group. Luminal bleeding was solely observed in one of 5 rats from indomethacin control group. The number of ulcer count was reduced to 0.1 ± 0.3 per rat in GSPEs treated group compared to 1.4 ± 0.5 per rat in indomethacin control group. Submucosal inflammatory cell infiltration was also reduced to 50% in GSPEs treated group. The tissue level of prostaglandin E_2_ was not affected by GSPEs treatment. GSPEs attenuated the indomethacin-induced small intestinal injury irrespective of the tissue PGE_2_ depletion and glutathione consumption.

## 1. Introduction

Nonsteroidal anti-inflammatory drugs (NSAIDs) have been shown to be effective and useful agents for a variety of diseases including rheumatic, musculoskeletal, and cardiovascular diseases. Despite the therapeutic benefits of NSAIDs, the occurrence of gastrointestinal toxicity is a major impediment for clinical utility [1, 2]. Compared to the stomach, the small intestine has been less appreciated as a target site of NSAID-induced gastrointestinal toxicity. However, with advances in diagnostic modalities for small intestinal disease, the concern about NSAID-induced small intestinal injury is growing, especially for the long-term NSAID users.

The pathogenesis of NSAID-induced intestinal injury is complicated and still not completely understood; NSAIDs act via both systemic and topical pathways. Systemic effects occur through inhibition of cyclooxygenase (COX); however, COX inhibition appears to play a smaller role in small intestinal injury than in gastric injury [3]. The topical toxicity of NSAIDs begins through direct interaction of NSAIDs with phospholipids in the cell membrane. Three crucial steps seem to be involved: (1) uncoupling of mitochondrial oxidative phosphorylation which leads to increased intestinal permeability; (2) attack of the mucosa by bile and luminal bacteria [4]; and (3) neutrophil chemotaxis and activation leading to mucosal inflammation. In addition, enterohepatic recirculation of NSAIDs is considered to enhance topical mucosal injury by repeated exposure of the intestinal mucosa to NSAID-concentrated bile [5]. Through these cascades, activated neutrophils and inducible nitric oxide synthase (iNOS) result in oxidative stress and produce reactive oxygen species (ROS) in the mucosa of the small intestine [6–8].

Proanthocyanidins, which belong to a class of flavonoid polyphenols, are widely distributed throughout the plant kingdom. Proanthocyanidin extracted from grape seed (GSPEs) acts as an antioxidant and has been reported to possess a broad spectrum of pharmacological and clinical properties against oxidative stress. GSPEs scavenge reactive oxygen free radicals and reduce intracellular oxidative status in* in vitro* experiments [9–11].

We hypothesized that, by scavenging free radicals, GSPEs would relieve oxidative stress in the small intestinal mucosa and reduce the injury induced by indomethacin. This study was designed to examine the protective effect of GSPEs on intestinal mucosal damage induced by acute administration of indomethacin in the rat.

## 2. Materials and Methods

### 2.1. Experimental Animals

Specific pathogen free Sprague-Dawley male rats (200 g) were purchased (Kostec, Pyeongtaek, Republic of Korea). They were bred in a restricted-access room with controlled temperature (20–24°C), humidity (40–60%), and 12-hour light/dark cycles. They were placed in stainless steel cages with wood chip bedding. A maximum of three rats were housed per cage. Food and water were provided* ad libitum*. All rats were acclimatized for four days before commencement of the experiment. This animal study was conducted in compliance with the Guide for the Care and User of Laboratory Animals issued by the American Institute of Laboratory Animal Resources.

### 2.2. Induction of Small Intestinal Lesions

**Figure 1:**
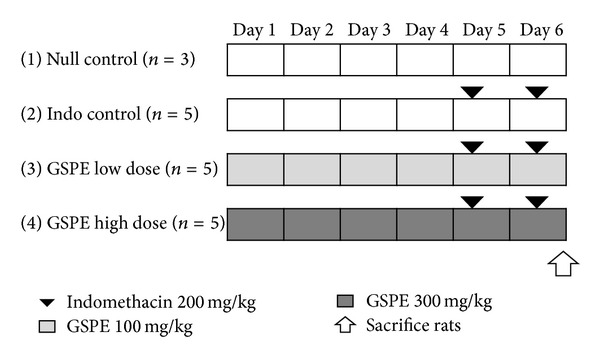
Study design. Rats were randomly allocated to one of four groups and treated with indomethacin and GSPE. Group 1: null control treated with carboxymethylcellulose solution (CMC) and saline; Group 2: indomethacin control treated with indomethacin at the dose of 200 mg/kg in 0.5% CMC; Group 3: GSPE low-dose group treated with GSPE in saline once daily at the dose of 100 mg/kg; Group 4: GSPE high-dose group treated with GSPE in saline once daily at the dose of 300 mg/kg. Indo: indomethacin; GSPEs: grape seed proanthocyanidin extracts.

Rats were allocated into four groups: three rats into the null control group, five rats into the indomethacin control group, five rats into the low-dose GSPEs group, and five rats into the high-dose GSPEs group. The study design is depicted in Figure 1.

Small intestinal mucosal injury was induced in both the indomethacin control group and the GSPEs treated groups by indomethacin (200 mg/kg of body weight) for two consecutive days. Indomethacin was prepared as a suspension in 0.5% carboxymethylcellulose at 40 mg/mL concentration and was administered by gavage under light anesthesia with ether. This dosage was chosen based on our preliminary study using 75 mg/kg, 100 mg/kg, and 200 mg/kg. Rats from the null control group received an equal volume of the vehicle (0.5% carboxymethylcellulose). At six hours after the last administration, rats were sacrificed by cervical dislocation under deep anesthesia with Zoletil; their abdomens were opened immediately and their small intestines were completely removed.

Proanthocyanidin, the flavonoid antioxidant, was prepared as grape seed proanthocyanidin extracts (GSPEs; Hanlim Pharm. Co., Ltd., Seoul, Republic of Korea) from* Vitis vinifera*. It is a procyanidolic oligomer chemically composed of a mixture of pycnogenol and flavonoid, with a structure of flavane-3-ol and the condensed polymers, including procyanidol dimer, trimer, and oligomer. To evaluate the effect of proanthocyanidin, GSPEs were administered to rats for four consecutive days by gavage and then coadministered one hour ahead of indomethacin for the last two consecutive days. Rats were administered with GSPEs at a dose of 100 mg/kg body weight or 300 mg/kg body weight according to assigned groups. Rats from the null and indomethacin control groups received saline at the same volume by gavage.

### 2.3. Macroscopic Evaluation

The small intestine was removed from each rat and the first 5.0 cm segment of duodenum and proximal jejunum and the last 5.0 cm segment of ileum were obtained. The intestinal segment was opened along the antimesenteric border, gently cleaned of luminal content, fixed on a plate, and photographed for macroscopic evaluation of mucosal damage. Mucosal damage was classified as perforation, presence of bleeding, and mucosal ulceration.

### 2.4. Histological Evaluation

**Figure 2:**
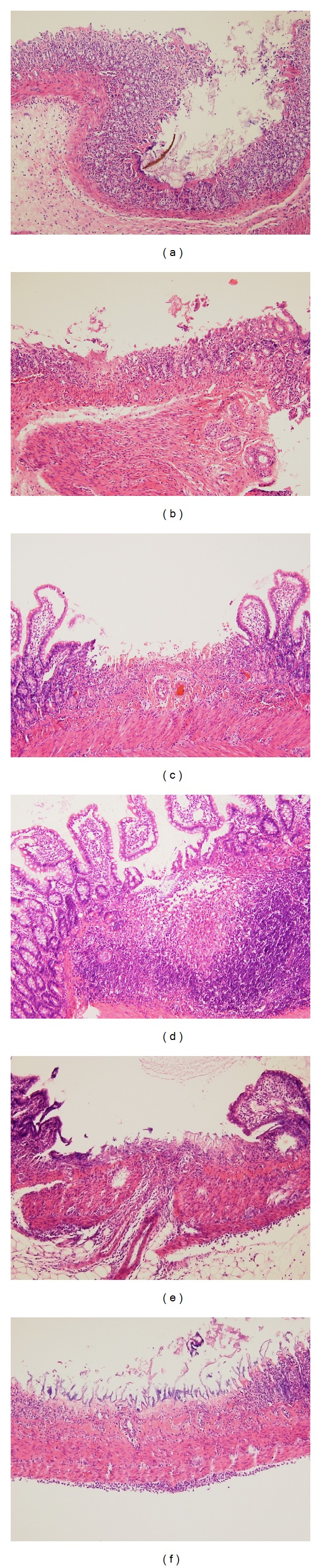
Microscopic findings in the rat small intestine. (a) and (b) Erosions induced by indomethacin showed focal villous necrosis and reduced epithelial height. (c) and (d) Ulcers induced by indomethacin showed deep excavation of mucosa and exposed submucosal layer. Marked inflammatory cell infiltration was noted in the submucosal space. (e) and (f) Transmural inflammatory infiltration was present beyond the ulcer formation (hematoxylin-eosin stain, ×40).

Histological evaluation was performed on the proximal 5.0 cm segment of duodenum and proximal jejunum and distal 5.0 cm segment of ileum. The intestinal segments were immediately immersed in 10% formalin solution and fixed for 24 hours. Formalin-fixed intestines were longitudinally sliced into 2.0 mm wide segments and processed into paraffin blocks. Mounted tissue slides were stained with hematoxylin-eosin. Histological findings of mucosal damage were assessed by an independent pathologist blinded to treatment, according to the following criteria (Figure 2):

- presence of submucosal neutrophilic infiltration:
   scanty, <10 neutrophils/HPF or
   obvious, ≥10 neutrophils/HPF,
- presence of erosion and loss of epithelial lining,
- presence of ulcer and exposure of submucosal tissue,
- transmural inflammatory infiltration along with ulcer.

Numbers of ulcer and erosion were counted per rat. With the assumption that the ulcer and erosion are shaped round, the total area of ulcer and erosion was calculated per rat.

### 2.5. Measurement of Tissue Prostaglandin E_2_ Levels

Intestinal tissue samples from resected intestine were immediately immersed in liquid nitrogen and then stored at −80°C. To obtain tissue homogenates, 1 mL homogenization buffer (0.1 M phosphate buffer, pH 7.4, containing 1 mM EDTA and 10 *μ*M indomethacin) per 100 mg of tissue was added. Homogenates were centrifuged at 8,000 g for 10 minutes and supernatants were collected. Determination of the tissue PGE_2_ level was performed using Cayman's PGE_2_ EIA kit–Monoclonal (Cayman Chemical, Ann Arbor, MI). The results are expressed in pg/mL and the lower limit for detection is 15 pg/mL.

### 2.6. Measurement of Tissue Glutathione Level

The resected intestine was rinsed with a phosphate buffered solution (pH 7.4) to remove any red blood cells and clots. To obtain tissue homogenates, 5–10 mL of cold buffer (i.e., 50 mM 2-(N—morpholino) ethanesulfonic acid, pH 7.0 containing 1 mM EDTA) per gram tissue was added to the tissue sample. Homogenates were centrifuged at 10,000 g for 15 minutes at 4°C. Supernatants were collected and deproteinated using 10% metaphosphoric acid and 4 M triethanolamine solution before analysis. Determination of the tissue glutathione level was performed using Cayman's GSH Assay kit (Cayman Chemical, Ann Arbor, MI). The GSH concentration of each sample was calculated by measuring the absorbance by 5-thio-2-nitrobenzoic acid (TNB) at 405 nm according to the manufacturer's instruction. The results are expressed in *μ*M/L.

### 2.7. Measurement of Serum IL-1*β*

Blood samples were obtained by cardiac puncture and collected directly into tubes containing EDTA. The collected blood samples were centrifuged immediately at 2,000 g for 15 minutes and plasma was stored at −20°C for later estimation of plasma IL-1*β*. Determination of serum IL-1*β* level was performed using specific kits based on an enzyme-linked immunosorbent assay (R&D Systems, Minneapolis, MN). The procedure was performed according to the manufacturer's instructions. IL-1*β* assay sensitivities were <3 pg/mL. The variability quotient was less than 15% in both assays. The results are expressed in pg/mL.

### 2.8. Statistical Analysis

The results were expressed as the mean ± standard deviation (SD). Differences among groups were evaluated by one-way analysis of variance and a *t*-test for numerical parameters. Differences for nonnumeric factors were compared by using Chi-square test. *P* values <0.05 were considered statistically significant.

## 3. Results

### 3.1. Macroscopic Damage

**Figure 3:**
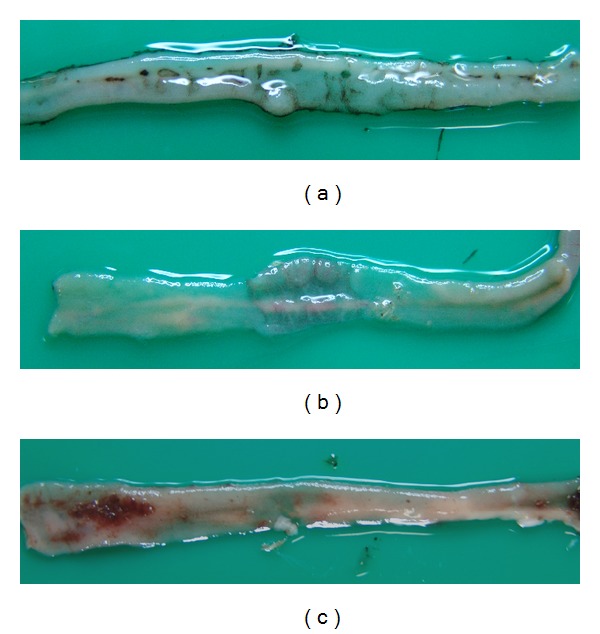
Gross appearance of small intestinal mucosal injury. (a) Multiple rounds, ovoid erosions, and ulcers were scattered in the small intestinal mucosa. Small erosions and ulcers measured from 0.1 mm to 1.5 mm in diameter. (b) A large ulcer created an 11.0 mm long circumferential band-like mucosal defect. The wall was thin and appeared transparent with impending perforation. (c) Spotty epithelial hemorrhages and bloody luminal content were present.

Small intestinal mucosal injury was induced by oral administration of indomethacin at the dose of 200 mg/Kg in rats. Mucosal injuries ranged from ulcers (Figure 3(a)) and thinning of the intestinal wall impending perforation (Figure 3(b)) to the presence of bloody content in lumen (Figure 3(c)). Small intestinal perforation was not found. Grossly discernable hemorrhage was observed in one rat from the indomethacin control group (Figure 3(c)). Macroscopic ulcers were observed in all rats from the indomethacin control group (*n* = 5) and in one from the GSPEs high-dose group (*n* = 5). No rats from the null control (*n* = 3) or GSPEs low-dose group (*n* = 5) showed ulcers in the intestine (*P* value = 0.02). Administration of GSPEs reduced the risk of ulcer development to 0.17 (*P* value = 0.001, 95% confidential interval; 0.028, 0.997) (Table 1).

### 3.2. Microscopic Damage

**Figure 4:**
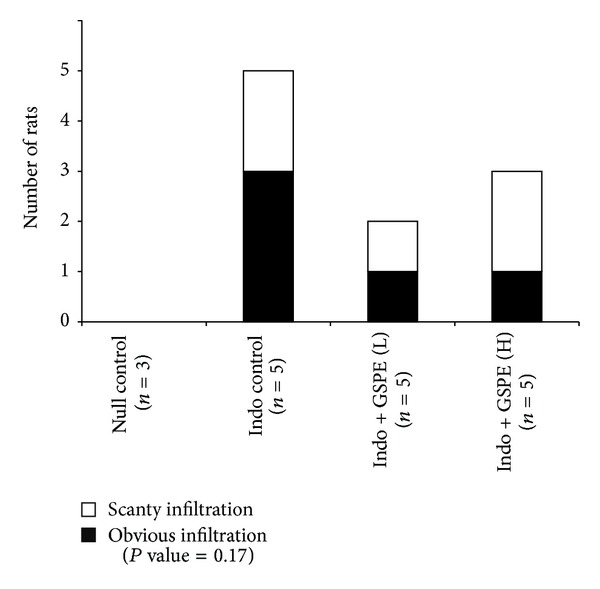
Histologic findings for inflammatory cell infiltration in submucosa. Submucosal inflammatory infiltration was present in all rats of indomethacin control group and about half of the rats in GSPE treated groups (*P* value = 0.17).

Submucosal inflammatory cell infiltration was graded as none, scanty, or obvious. In the indomethacin control group, submucosal inflammatory infiltration was observed in all rats. Obvious submucosal inflammatory infiltration was present in three rats (60%) and scanty infiltration was in two rats (40%). In the GSPE low-dose group, three rats (60%) showed no submucosal inflammatory infiltration. Obvious and scanty infiltration patterns were observed in one rat each. In GSPEs high-dose group, two rats (40%) showed no submucosal inflammatory infiltration. Obvious and scanty infiltration patterns were observed in one and two rats, respectively (*P* value = 0.17) (Figure 4). In sum, submucosal inflammatory cell infiltration was observed in all rats from indomethacin control group and in 50% of rats from GSPEs treated group (*P* value = 0.019). According to submucosal inflammatory infiltration, there was no difference between the GSPE high- and low-dose groups.

**Figure 5:**
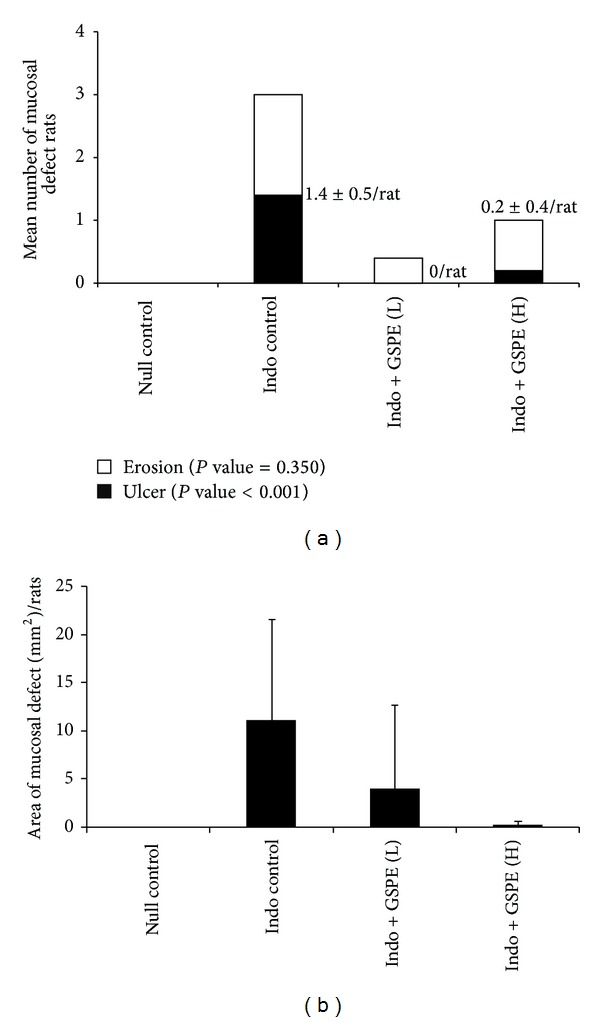
Areas and numbers of ulcers and erosions. (a) The mean number ± SD of ulcers was 1.4 ± 0.5/rat in the indomethacin control group, zero in the GSPE low-dose group, and 0.2 ± 0.4/rat in the GSPE high-dose group (*P* value <0.001). The number of erosions was not significantly different among the treatment groups. (b) The mean area ± SD of mucosal defects was 11.1 ± 10.5 mm^2^/rat in the indomethacin control group, 3.9 ± 8.7 mm^2^/rat in the GSPE low-dose group, and 0.3 ± 0.4 mm^2^/rat in the GSPE high-dose group.

More severe mucosal injury by indomethacin administration presented as erosions and ulcers. The number of ulcers per rat varied according to whether GSPEs administration was given (*P* value <0.001). The number of ulcers per rat was 1.4 ± 0.5 for the indomethacin control group and 0.2 ± 0.4 for the GSPEs high-dose group. None of the rats from the null control group or the GSPEs low-dose group had ulcers. Compared to the indomethacin control group and rats treated with GSPEs, the number of ulcers per rat was significantly higher in the indomethacin group (1.4 ± 0.5 versus 0.1 ± 0.3, *P* value = 0.004). The number of erosions per rat was 1.6 ± 2.2 for the indomethacin control group, 0.4 ± 0.5 for the GSPEs low-dose group, and 0.8 ± 0.8 for the GSPEs high-dose group (*P* value = 0.35) (Figure 5(a)). There was no difference between the GSPEs high- and low-dose groups.

Assuming each lesion was rounded in shape, the calculated area of the mucosal defect was also higher in the indomethacin control group. The total area of the mucosal defect, including ulcers and erosions, was 11.1 ± 10.5 mm^2^ per rat for the indomethacin control group. For the GSPEs low-dose group, the area of the mucosal defect was reduced to 4.0 ± 8.7 mm^2^ and it was 0.3 ± 0.4 mm^2^ for the GSPEs high-dose group (*P* value = 0.13) (Figure 5).

Transmural inflammatory infiltration was noted in three subjects, all of which were in the indomethacin control group (*P* value = 0.03).

Regarding the location within the intestine, the severity of mucosal injury was not different between the proximal and distal segments of the small intestine. The total numbers of ulcers and erosions were 0.7 ± 0.9 and 0.6 ± 1.2 for the proximal and distal segments, respectively (*P* value = 0.76).

### 3.3. Tissue PGE_2_ and Glutathione Levels

**Figure 6:**
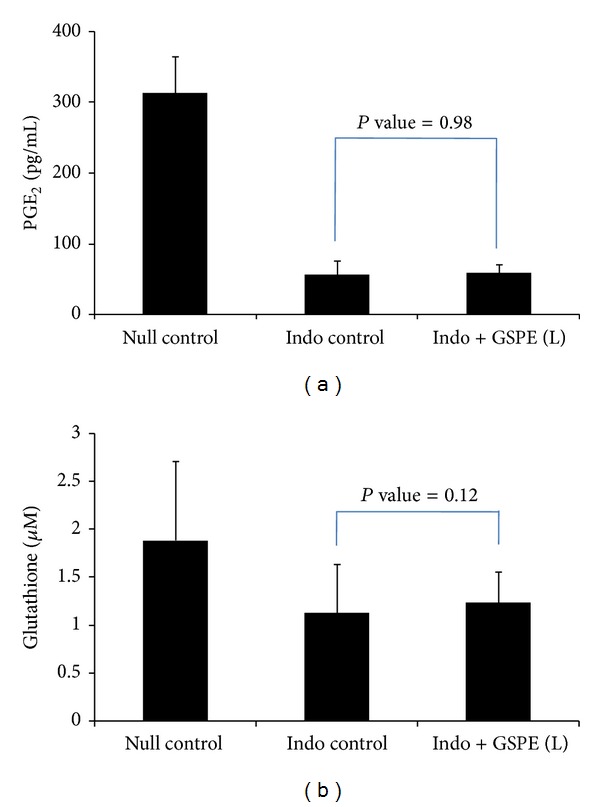
Tissue level of PGE_2_ and glutathione. (a) The level of PGE_2_ was 313.5 ± 51.9 pg/mL for the null control group, 55.6 ± 20.3 pg/mL for the indomethacin control group, and 58.6 ± 11.6 pg/mL for the GSPE low-dose group (*P* value = 0.983). (b) The tissue glutathione concentration was 1.98 ± 0.83 *μ*M for the null control group, 1.13 ± 0.51 *μ*M for the indomethacin control group, and 1.24 ± 0.32 *μ*M for the GSPE low-dose group (*P* value = 0.12).

The tissue PGE_2_ level was 313.5 ± 51.9 pg/mL for the null control group, 55.6 ± 20.3 pg/mL for the indomethacin control group, and 58.6 ± 11.6 pg/mL for the GSPEs low-dose group (*P* value = 0.983) (Figure 6). The tissue glutathione concentration was 1.98 ± 0.83 *μ*M for the null control group, 1.13 ± 0.51 *μ*M for the indomethacin control group, and 1.24 ± 0.32 *μ*M for the GSPEs low-dose group (*P* value = 0.12).

### 3.4. Serum IL-1*β*

Serum IL-1*β* was measured by using a specific ELISA kit and the values did not vary between treatments: zero for the null control group, 10.5 ± 21.0 pg/mL for the indomethacin control group, 12.6 ± 18.0 pg/mL for the GSPEs low-dose group, and 12.6 ± 28.2 pg/mL for the GSPEs high-dose group (*P* value = 0.82) (Figure 6).

## 4. Discussion 

Clinical and practical studies of NSAID-induced intestinal toxicity have been outside the focus of most major researches. As the aged population grows, the number of chronic NSAID users continues to rise, and NSAID-induced small intestinal injury is encountered more frequently than before. Small intestinal injury resulting from NSAID use includes bleeding from ulcers and erosions, stricture or stenosis of the lumen, perforation of the wall, protein losing enteropathy, and aggravation of preexisting inflammatory bowel diseases [12, 13]. To prevent small intestinal injuries caused by NSAIDs, several agents have been tried in experimental animal studies [7, 14–16] and human studies [17]. These agents can be categorized into two groups based on their mode of action: prostaglandin analogues [14] and antioxidants [7, 15–17]. The pathogenesis of NSAID-induced small intestinal injury is different from that of gastric injury. In addition to inhibition of COX and prostaglandin synthesis, oxidative stress due to mitochondrial dysfunction and inflammatory cascades play crucial roles in small intestinal mucosal injury [3, 18]. COX-1 is a well-known constitutive enzyme in the gastrointestinal mucosa that is responsible for prostaglandin synthesis. NSAIDs inhibit COX-1 and thereby deplete prostaglandin in tissues which mediate mucosal bicarbonate production, mucus secretion, and maintenance of blood flow. COX-2 is an inducible enzyme in inflammatory conditions that is responsible for pyretic and painful responses. Recent efforts to avoid the gastrointestinal toxicity induced by NSAIDs have been aimed at developing an agent that selectively inhibits COX-2. COX-2 selective inhibitors (CSIs) including rofecoxib have a 50% reduced risk of upper gastrointestinal toxicity, compared to nonselective inhibitors such as naproxen [19, 20]. However, long-term COX-2 selective inhibition still leads to serious gastrointestinal toxicity, and CSIs raise the risk of cardiovascular events such as myocardial infarction, stroke, and elevated blood pressure. In the small intestine, there are additional roles for COX-2. COX-2 in small intestinal mucosa acts as an immune modulator inducing oral tolerance and maintaining homeostasis with gut flora in the small intestine, and it participates in the mucosal healing process. COX-2 inhibition with normal COX-1 activity resulted in disruption of epithelial integrity and mucosal injury in an experimental study [21]. Therefore, COX-2 selective inhibition is not as safe or as tolerable as expected, especially in the small intestine. Oxidative stress from mitochondrial dysfunction and inflammatory cascades is initiated by topical injuries caused by NSAIDs. NSAIDs, as weak acids, freely pass through and interact with phospholipids in cell membrane. Ionized NSAIDs, trapped in the cytoplasm, cause biochemical damage to mitochondria and uncoupling of oxidative phosphorylation [6, 7]. Cellular oxidative stress results in disruption of epithelial integrity and translocation of bacteria and luminal toxic agents [3, 22]. This leads to intestinal mucosal inflammation including neutrophilic tissue infiltration. Oxidants including the superoxide radical and hydrogen peroxide are produced by inflammatory cells. Nitric oxide (NO) from inducible nitric oxide synthase (iNOS) reacts with superoxide and is converted into peroxynitrite and then into reactive hydroxyl radicals [23]. These free radicals and the superoxide anion destroy DNA and cause cellular damage, particularly injuring villous tip cells, causing functional and structural impairment of the brush border membrane [24, 25]. Experiments with epithelial cell lines showed an involvement of oxygen free radicals in epithelial damage and dysfunction and demonstrated reversal of the damage with antioxidant pretreatment [26].

Antioxidant systems in the body include superoxide dismutase, glutathione peroxidase, glutathione reductase, vitamin E, and vitamin C. Antioxidants suppress excessive oxidative stress by reacting with free radicals and scavenging ROS. GSPE from* Vitis vinifera* is an antioxidant that acts against oxidative stress by radical-scavenging, quenching, and enzyme-inhibiting actions. At a 100 mg/liter concentration, GSPEs exhibit 78–81% inhibition of the superoxide anion and hydroxyl radical and show scavenging ability comparable to that of the combination of superoxide dismutase and catalase [10].* In vitro*, GSPEs showed a concentration-dependent amelioration of the intracellular oxidized state induced by hydrogen peroxide modulation [11]. In* in vivo* model of NSAID-induced gastric injury, GSPEs proved protective effects by reducing oxidative stress in tissue level [9]. Therefore, GSPE should be protective against small intestinal mucosal injury in which free radical production and tissue antioxidant depletion play crucial roles in cell and tissue damage.

The usual dose of indomethacin for clinical practice is 200 mg/day or less for adult human. This is about 3 mg/Kg for a man with 60 Kg weight. In this experiment, we adopted rat model and literatures recommended about 50 mg/Kg to induce intestinal mucosal injury regardless of administration route. This is over 15-times high dose compared to doses for human. However, authors had failed to find mucosal injury at 50 mg/Kg dose at initial pilot experiment. Authors tried various doses in subsequent preliminary study, including 75 mg/kg, 100 mg/kg, and 200 mg/kg. Finally, the dose of 200 mg/Kg for 2 days was successful to induce small intestinal mucosal injury and was chosen for this study. Authors supposed that 2 days of indomethacin administration is relatively short period to induce significant intestinal mucosal injury with the dose of 50 mg/Kg.

At a dose of indomethacin 200 mg/kg of body weight, small intestinal injury was induced in rats of indomethacin control group. Macroscopic ulcers and bloody luminal contents, suggestive of bleeding, were observed as expected. Histologically, inflammatory cell infiltration was present in the submucosa of all rats and expanded to the muscle proper layer in a severe case. The COX expression of intestinal epithelium is one of the important factors in the process of NSAID-induced intestinal injury. To assess the inhibitory effect of indomethacin on COX, we measured the tissue level of PGE_2_ in the small intestinal mucosa. The level of PGE_2_ in indomethacin-administered rats was markedly lowered compared to that in rats from the null control group. The level of tissue glutathione was measured in the small intestinal mucosa to assess the status of tissue antioxidant consumption which inversely represents the degree of oxidative stress state. In rats administered indomethacin, the level of tissue glutathione was reduced, suggesting elevated oxidative stress in the tissue.

In rats administered with GSPEs and indomethacin, small intestinal mucosal injury was significantly less than in rats administered indomethacin alone. The frequency of small intestinal mucosal ulcers was significantly less in rats administered with GSPEs. The submucosal inflammatory infiltration was found less frequently in the small intestine of rats administered with GSPEs, and transmural infiltration was not observed in any rat. The level of tissue PGE_2_ declined markedly in rats administered with GSPEs and was comparable to that in rats administered indomethacin alone. These suggested that GSPEs did not affect COX inhibition by indomethacin. Based on these results we inferred that PGE_2_ depletion by COX inhibition plays only a partial role in the development of small intestinal mucosal injury in rats administered indomethacin; furthermore, there should be another branch in the mucosal injury pathway that is reversed by GSPEs. Even though the difference between the levels of glutathione in tissues from the indomethacin control and GSPEs low-dose group was not statistically significant, there was a tendency toward reduced consumption of glutathione in rats from the GSPEs low-dose group. The systemic inflammatory response as measured by serum IL-1*β* showed no difference among the groups. This suggests that the small intestinal mucosal injury due to indomethacin is a locally confined inflammatory reaction.

Limitations exist in our study. The GSPEs are crude extracts of grape seed which contain proanthocyanidin as a key component. Other minute materials in crude extract may exert a role in injury and protection of the study protocol. Though purified material or synthetic pure chemical may result in more defined influence in animal model experiment, authors cannot help but use commercially available preparation of GSPEs. Another limitation lies in the duration of NSAID administration. In clinical practice, NSAID related intestinal complications often occur following long-term or chronic administration. Long standing administration of NSAID can alter the physiology of cell and COX expression level. In this study, authors made intestinal injury with 2 days of high dose indomethacin. There possibly is a discrepancy between this experiment and real clinical practice.

In summary, our results show that proanthocyanidin protects the small intestinal mucosa of rats from the injurious effects of indomethacin. This protective effect of proanthocyanidin is suggestive to be related to a reduced tissue oxidative stress and related neither to the depletion of tissue PGE_2_ nor to the systemic inflammatory reaction. Further studies should be carried out to determine whether proanthocyanidin's antioxidant activity and associated protective effects are applicable to humans and whether this agent could be used as a novel therapeutic drug to prevent the NSAID-induced small intestinal injury.

## Figures and Tables

**Table 1:** The incidence of indomethacin-induced small intestinal ulcers.

	Null control	Indo control	Indo + GSPE (L)	Indo + GSPE (H)	*P* value
	(*n* = 3)	(*n* = 5)	(*n* = 5)	(*n* = 5)	
Ulcer	0	5 (100%)	0	1 (20%)	0.02
Blood in lumen	0	1 (20%)	0	0	

Indo: indomethacin; GSPEs (L): grape seed proanthocyanidin extracts low dose 100 mg/kg; GSPEs (H): grape seed proanthocyanidin extracts high dose 300 mg/kg.
